# Case report: Minimally invasive management of synchronous early-stage ascending colon adenocarcinoma and type 1 papillary renal cell carcinoma presenting with severe anemia: a rare Chinese case

**DOI:** 10.3389/fonc.2025.1698127

**Published:** 2026-01-05

**Authors:** Jiahao Li, Kai Guo, Li Yi, Chenrong Yuan, Ningjuan Tang, Simei Wu, Yongdong Zhu, Yue Tan, Zhi Wang, Wanyong Yang, Yuanzan Zhu, Yu Tian, Sangui Wang, Quanlei Wang

**Affiliations:** 1 Department of Surgery, Dongguan Nancheng Hospital, Dongguan, China; 2 Department of Pathology, Dongguan Nancheng Hospital, Dongguan, China; 3 Department of Internal Medicine, Dongguan Nancheng Hospital, Dongguan, China; 4 Department of Oncology, Dongguan Nancheng Hospital, Dongguan, China; 5 Department of Radiology, Dongguan Nancheng Hospital, Dongguan, China; 6 Department of Laboratory, Dongguan Nancheng Hospital, Dongguan, China; 7 Clinical Trial Research Organization, Dongguan Nancheng Hospital, Dongguan, China; 8 Dongguan Institute of Gallbladder Disease Research, Dongguan Nancheng Hospital, Dongguan, China

**Keywords:** anemia, synchronous, ascending colon adenocarcinoma, type 1 papillary renal cell carcinoma, laparoscopic surgery

## Abstract

**Background:**

Ascending colon adenocarcinoma(ACA) synchronous with papillary renal cell carcinoma (pRCC) is extremely rare, with only sporadic cases reported in the literature around the world. The oncogenic mechanisms, early diagnosis, and minimally invasive treatment of the coexistence of ACA and pRCC face significant challenges. This article describes a case diagnosed with concurrent ACA and pRCC at early-stage presented with severe anemia were successfully treated in a primary care centers in China.

**Case presentation:**

A 66-year-old male with abdominal distension, fatigue, and weakness lasting over 1 month was presented to our outpatient clinic. Laboratory tests revealed severe anemia, while imaging examination were highly suspicious for both colon cancer and renal carcinoma. Colonoscopy confirmed the presence of a mass in the ascending colon, and the biopsy results suggested adenocarcinoma. Then, after a multidisciplinary discussion, blood transfusion and iron therapy were applied to improve the anemia, then the patient underwent laparoscopic radical right hemicolectomy and left kidney lesion resection surgery under general anesthesia. Postoperative pathology revealed ACA and type 1 pRCC. The patient subsequently received postoperative chemotherapy etc, and the prognosis was favorable.

**Conclusion:**

Synchronous ACA with type 1 pRCC are extremely rare. The early stage diagnosis and minimally invasive treatment of the concurrent ACA and pRCC was still challenges. Preoperative anemia improvement, laparoscopic tumor resection combined with postoperative chemotherapy etc is an important treatment strategy for early-stage ACA and pRCC, as it allows for the simultaneous radical resection of tumors in both locations. The multidisciplinary management model is crucial for the personalized treatment of patients with complex malignancies in primary care center.

## Introduction

Colorectal cancer is one of the most common cancers in the world, however, the simultaneous presence of other neoplasms such as renal cancer is rare. Most colon and renal carcinomas were metachronous. Based on our literature review, cases of synchronous ascending colon adenocarcinoma(ACA) and papillary renal cell carcinoma (pRCC) are exceedingly rare, sporadic cases were reported, with the majority reported in Western populations (1). The representation of Asian, and specifically Chinese, patients in the literature remains notably scarce. The oncogenic mechanisms, early diagnosis, and minimally invasive treatment of the coexistence of ACA and pRCC face significant challenges (2).

In this study, we present a rare case of a Chinese patient with synchronous colon cancer and renal cell carcinoma. The patient underwent minimally invasive laparoscopic surgery to simultaneously resect tumors from both organs, combined with acupuncture, chemotherapy, etc adjunctive supportive care and has currently achieved a satisfactory clinical outcome.

## Case report

### Preoperative comprehensive examination

The patient with abdominal distension, fatigue, and weakness lasting over 1 month was presented to our outpatient clinic, and had no history of any disease or surgery. The patient’s mental state was poor and his skin appeared pale; however, there were no signs of jaundice or cardiopulmonary abnormalities. The vital signs were as follows: temperature, 36.7°C; pulse, 90/min; and blood pressure, 103/62 mmHg, BMI, 23cm/kg. The patient’s abdomen was flat, without tenderness noted in both the right upper and right lower quadrants. He denied familial neoplasm.

Laboratory tests indicated the patient’s hemoglobin was 46.0g/L, red blood cell count was 2.61x10^12/L; Creatinine level was 171umol/L, β2-microglobulin level was 4.45mg/L, uric acid level was 483umol/L; Iron determination level was 5.4umol/L, transferrin saturation level was 8.5%; White blood cell count was 10.34x10^9/L, hypersensitive C-reactive protein: 25.11mg/L. Urine analysis demonstrated microhematuria: 3+ and protein:1+. Tumor markers were normal (CA125; CA153) except for CEA, which was at 25.62 ng/mL. The above results indicated that the patient has severe anemia and kidney impairment, accompanied by an inflammatory response.

**Figure 1:**
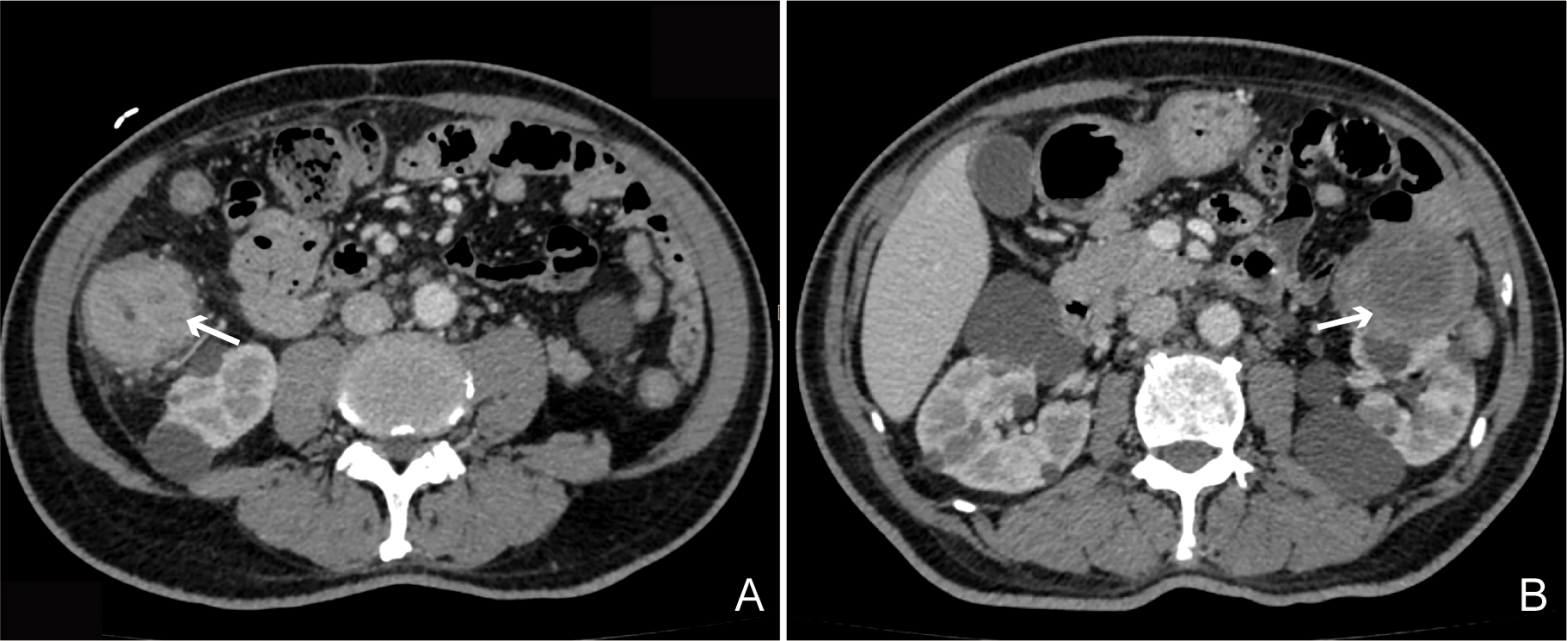
The abdominal CT scan demonstrating the presence of an ascending colon mass **(A)** and solid renal mass **(B)**.

**Figure 2:**
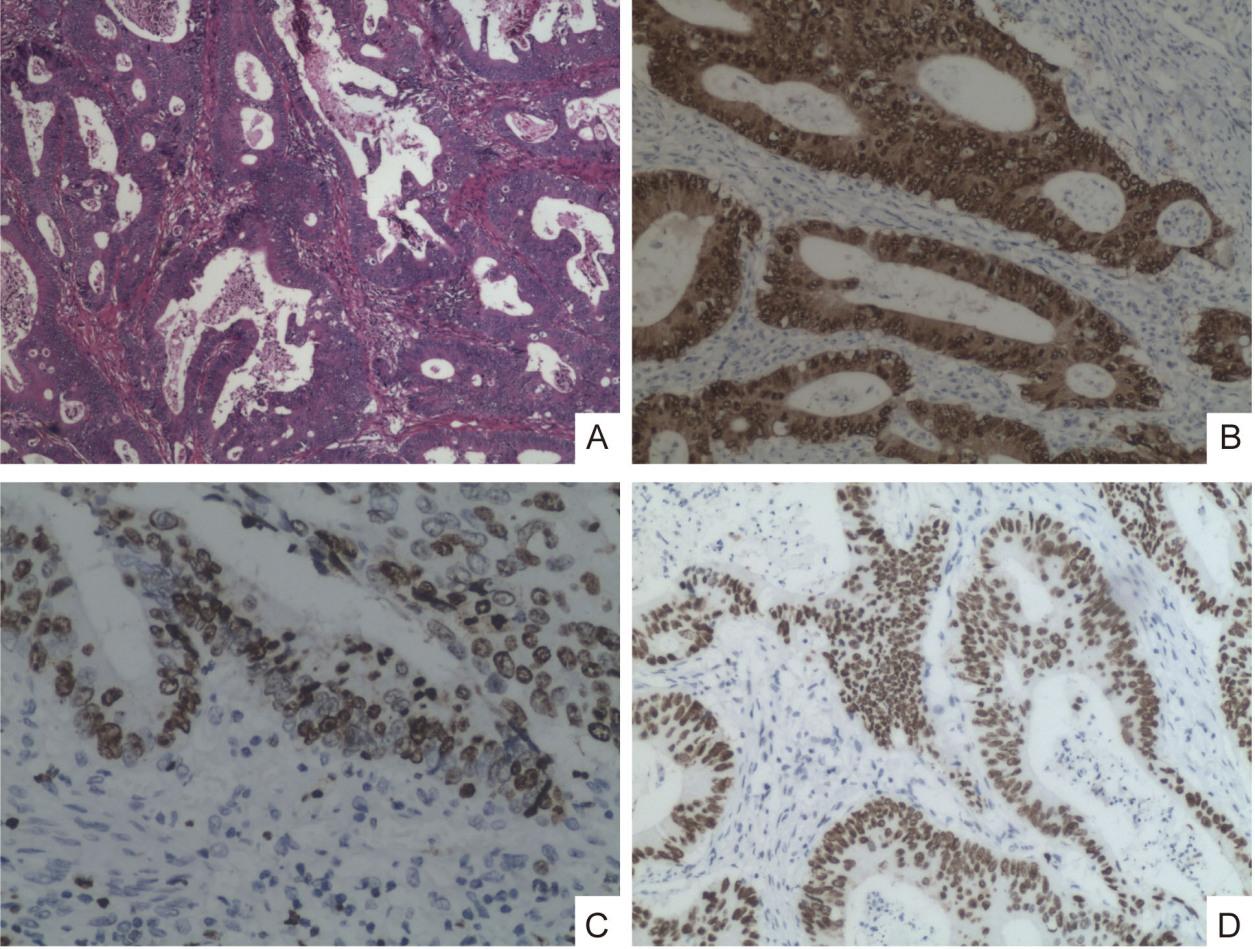
Low power view showing the colonic mucosa is infiltrated by carcinomatous proliferation, primarily exhibiting tubular and cribriform patterns (Magnification ×4) **(A)**; Tumor cells showed nuclear expression of CDX2 (Magnification ×20) **(B)**; Tumor cells showed nuclear expression of Ki67 (Magnification ×20) **(C)**; Tumor cells nuclear expression of P53 (Magnification ×10) **(D)**.

The abdominal CT scan results reveal significant wall thickening in the hepatic flexure of the right colon, suggestive of colon cancer with perifocal fat infiltration; and a left renal mass, considered likely to be renal cell carcinoma, respectively (**Figures 1A, B**). The following day, the gastroenterology department performed a colonoscopy with biopsies, revealing a tubular stenotic lesion in the ascending colon near the hepatic flexure. The surface of the lesion showed exudate and small nodules, was friable, and had well-defined borders. The lumen was narrowed to the point that the endoscope could not pass through. Histological analysis of the biopsy showed the presence of a moderately differentiated adenocarcinoma with an immunohistochemical profile (CDX2+, Ki67+ and P53+) (**Figures 2B–D**).

### Minimally invasive laparoscopic surgery

Through multidisciplinary discussion, the patient received preoperative symptomatic treatment including blood transfusion and iron supplementation, and all relevant surgical contraindications were ruled out. After thorough discussion of all treatment options with the patient, the patient underwent laparoscopic minimally invasive radical right hemicolectomy and left renal lesion resection at the same surgery.

After successful anesthesia, the patient was placed in the right lateral decubitus position. The skin was routinely disinfected with povidone-iodine and covered with sterile drapes. Approximately 2 cm above the upper border of the left iliac crest and laterally, transverse incisions of about 1 cm were made respectively. A 1 cm trocar sheath was inserted through each incision via puncture. A laparoscope was introduced to dissect the retroperitoneal space, and CO_2_ gas was insufflated to establish an operative space. Punctures were performed along the anterior and posterior axillary lines below the costal margin to insert 0.5 cm trocar sheaths, establishing operative channels. The retroperitoneal space was dissected toward the peritoneum. Using an ultrasonic scalpel, the dissection continued upward along the peritoneum to the upper pole of the left kidney, and then further downward to the lower pole and renal hilum. A mass was observed protruding from the surface at the midportion of the left kidney. The renal pedicle vessels were dissected and isolated at the renal hilum, and a non-crushing vascular clamp was applied to occlude the renal pedicle vessels. The mass was completely excised and sent for pathological examination. The abdominal fluid was aspirated, a drainage tube was placed, and the incisions were sutured.

The patient was then repositioned supine. Exploration of the abdominal cavity identified a mass near the hepatic flexure of the ascending colon. The procedure began by entering the gastrocolic ligament on the right side and incising it to expose the gastrocolic trunk. Dissection proceeded along the lateral aspect of the duodenum towards the hepatic flexure. Subsequently, dissection continued along the ileocolic vessels towards the root of the mesenteric vessels. The ileocolic artery and vein, and the right colic artery and vein were ligated and divided. An accessory right colic vein and the right branch of the middle colic artery and vein were also dissected, ligated, and divided. The right peritoneum was incised, and dissection proceeded cephalad along the Toldt’s fascia plane until meeting the previous dissection from the cranial aspect. Proximally, the ileum was mobilized and transected approximately 15 cm from the ileocecal valve. Distally, the colon was mobilized and transected to the right of the middle colic artery. An intracorporeal side-to-side ileocolonic anastomosis was performed. The enterotomy was closed with 3.0 sutures. A drain was placed, and the incisions were closed.

**Figure 3:**
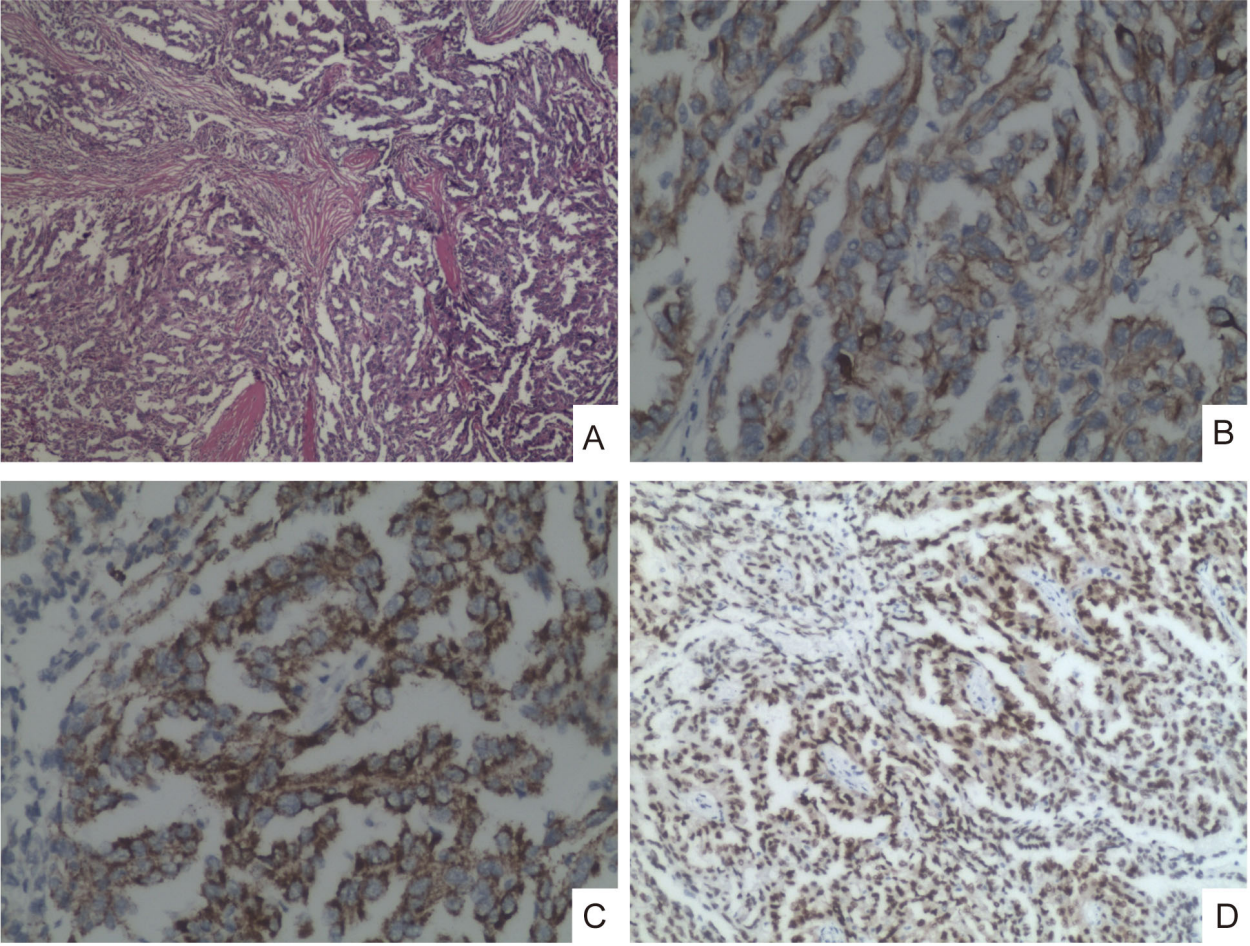
Under low-power view (4x magnification), the tissue exhibits infiltration by type 1 papillary renal cell carcinoma with predominant papillary patterns **(A)**. Tumor cells demonstrate cytoplasmic positivity for CK (20x magnification) **(B)**; Cytoplasmic staining for P504S (20x magnification) **(C)**; And nuclear reactivity for PAX8 **(D)** (10x magnification).

The surgery was completed successfully. Total intraoperative blood loss was approximately 400 mL, and urine output was 1000 mL. The total operative time was 3 hours and 10 minutes. Postoperative pathological findings were as follows: for the ascending colon cancer: the tumor size is 6.0 cm x 5.0 cm x 4.0 cm, ulcerative moderately differentiated adenocarcinoma of the ascending colon infiltrated the periintestinal adipose tissue, blood vessels, and nerves with no lymph node metastasis (0/16). Pathological TNM staging: T4aN0M0. For the renal cell carcinoma, the tumor size is 7.0 cm x 5.0 cm x 4.0 cm papillary structures centered by a thin conjunctiva-vascular axis with the presence of a psammomatous body. The positive for cancer cell markers were CK(+), P504S(+), and PAX8(+) (**Figures 3B–D**). Pathological TNM staging: T2aN0M0. The above results indicated synchronous early-stage ascending colon adenocarcinoma and type 1 papillary renal cell carcinoma in the presented patient.

### Adjunctive supportive care

Postoperative conventional treatment included symptomatic and supportive care such as antibiotics (anti-infection), stomach protection, renal protection, fluid replacement, and nutritional support. Postoperatively, bowel sounds were absent. Electroacupuncture supportive therapy was administered within the first 24 hours post-surgery to promote intestinal peristalsis based on previous study (3). Bowel sounds gradually returned within 48 hours postoperatively. No other significant complications occurred. The patient recovered well and was discharged two weeks after the surgery. He was subsequently undergo chemotherapy in our hospital. According to the National Comprehensive Cancer Network (NCCN) guidelines and multidisciplinary team discussion, the chemotherapy regimen was initiated as adjuvant treatment for Stage II colon cancer with high-risk features. The prescribed CapeOx (also known as the XELOX regimen) is planned for a total of 8 cycles. For Stage II renal cell carcinoma, current guidelines recommend considering clinical trials as the primary option, as no established chemotherapy regimens are available; management otherwise emphasizes regular surveillance and observation. The patient has currently completed 5 cycles of CapeOx smoothly with stable disease.

## Discussion

Primary tumors can be diagnosed simultaneously or metachronous depending on the time of diagnosis. Simultaneously primary tumors is defined by two or more neoplasms that developed within the same period or up to 6 months after the first diagnosis (4). Moreover, they must present different malignancies, different histological types and the possibility of one being a metastasis of the other must be excluded. Metachronous tumors are those that are evidenced between 6 months and three years. Globally, the sporadic reported cases of colon and renal cancer are either metachronous or metastatic tumors (5). These cases have progressed to an advanced stage at the time of diagnosis, making them no longer eligible for surgical resection (1). Moreover, the representation of Asian, and specifically Chinese, patients in the literature remains notably scarce. The oncogenic mechanisms, early diagnosis, and minimally invasive treatment of the coexistence of ACA and pRCC face significant challenges (2).

Although the diagnosis of ascending colon cancer is relatively straightforward, the early simultaneous detection of both cancers is exceedingly challenging. Existing reports on synchronous cancers indicate that patients often present at advanced stages and treatment is primarily palliative (6). In this case, the patient was identified through a national annual community health screening program for the elderly, which detected elevated CEA levels and decreased hemoglobin. This prompted further hospital investigation, leading to a definitive diagnosis of early-stage synchronous ascending colon cancer and papillary renal cell carcinoma through a combination of laboratory tests, imaging studies, and colonoscopy. In conclusion, the future expansion of population-wide annual screenings and tumor screening programs in grassroots healthcare institutions is of paramount importance for the early diagnosis of diseases, particularly multiple cancers (7).

Cancer development is regulated by a combination of genetic and environmental factors, and the occurrence of multiple primary cancers in an individual often has an underlying genetic basis, such as Lynch syndrome or, occasionally, sporadic events (8). Previous studies have found that synchronous and metachronous primary colon cancers and other malignancies may share common genetic backgrounds (9). With the rapid advancement of multi-omics technologies—including genomics, transcriptomics, epigenomics, and proteomics further integration with animal models to deeply investigate the genetic mechanisms of synchronous ascending colon cancer and papillary renal cell carcinoma will be highly significant for advancing clinical precision diagnosis, treatment, and prognosis (10).

With advances in medical technology, open surgery is increasingly less used for radical resection of early-stage tumors, and has been gradually replaced by minimally invasive approaches for the removal of both synchronous and metachronous multiple tumors (11, 12). In this case, through preoperative multidisciplinary discussion, the patient’s symptoms such as anemia were improved, surgical contraindications were strictly excluded, and laparoscopic resection of the lesions was performed. A single procedure achieved radical resection of tumors in two organ sites, fulfilling the goal of individualized precision medicine, while reducing medical resource utilization and patient hospitalization costs. satisfactory surgical outcomes were obtained postoperatively. Previous research has suggested that a simultaneous laparoscopic surgical approach for multiple carcinomas is associated with an increased risk of perioperative complications due to complex anatomy and diverse organ function (12, 13). It is crucial that a personalized surgical and postoperative care plan was formulated by the multidisciplinary team based on the cancer patient’s diagnostic findings (14). Recent clinical studies have indicated that acupuncture can facilitate rapid recovery of bowel function in cancer patients following surgery, particularly those with colorectal, gastric, and bladder cancers etc (15, 16). This adjuvant therapeutic approach holds significant clinical implications for postoperative rehabilitation in oncologic patients around the world (17–19). In the presented study, the patient recovered without major complications, and traditional Chinese medicine electroacupuncture was administered to address initial poor gastrointestinal motility, effectively promoting recovery by regulating intestinal activities to relieve bloating and constipation which were in line with previous study (3, 20). In summary, a physician’s profound knowledge of human anatomy and the effective leverage of multidisciplinary resources are crucial for personalized precision medicine. This case report also serves as a valuable reference for managing similar clinical scenarios in the future.

## Conclusion

The early stage precise diagnosis and minimally invasive treatment of the concurrent ACA and pRCC was still challenges. Preoperative anemia improvement, laparoscopic tumor resection combined with postoperative chemotherapy and multidisciplinary supportive treatment are an important treatment strategy for early-stage ACA and pRCC. A profound understanding of human anatomy and interdisciplinary collaboration are the cornerstones of personalized precision medicine for complex cancer patients in primary healthcare institutions.

## Data Availability

The original contributions presented in the study are included in the article/supplementary material. Further inquiries can be directed to the corresponding authors.
